# Supplementary material for: Microphthalmia-Associated Transcription Factor-Dependent Melanoma Cell Adhesion Molecule Activation Promotes Peritoneal Metastasis of Ovarian Cancer
Source: Int J Mol Sci. 2020 Dec 21;21(24):9776. doi: 10.3390/ijms21249776 (PMC7767511; doi:10.3390/ijms21249776)
Supplement: Supplementary file 1 [file ijms-21-09776-s001.pdf]

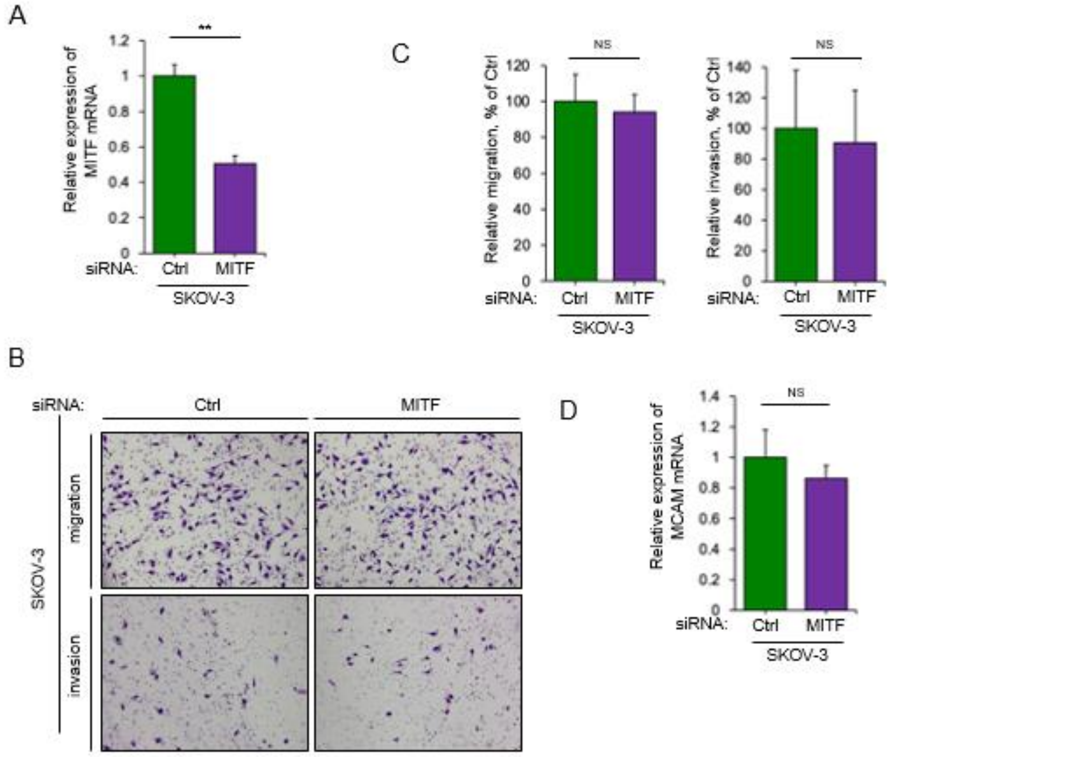

Supplementary Figure S1: MITF expression is not associated with motility and invasion of SKOV-3 cells. A) Relative expression levels of MITF mRNA in SKOV-3 cells under siRNA treatment. B) Representative images in the motility and invasion assays. C) Relative migration and invasion percentages in SKOV-3 cells under siRNA treatment. D) Relative expression levels of MCAM mRNA in SKOV-3 cell under MITF siRNA treatment.

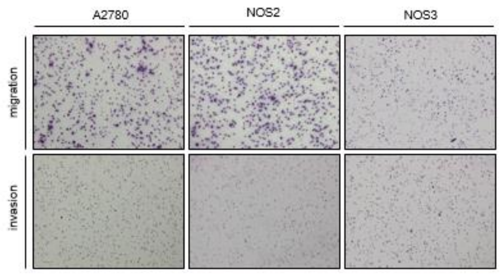

Supplementary Figure 2: MITF-low OvCa cells does not show motility and invasion abilities. Representative images in the motility and invasion assays with A2780, NOS2 and NOS3 cells.

Supplementary Table S1: Clinical characteristics of the patients used in the Immunoblot analysis.

patient	age	stage (FIGO2014)	TNM	operation	metastasis	NAC
2	77	IA	T1aN0M0	ATH + BSO + OM	-	-
36	50	IIIC	T3cN0M0	ATH + BSO + OM	peritoneal metastasis	-
44	45	IIIA1(ii)	T2bN1M0	ATH + BSO + OM	para aortic lymph node	-
50	56	IIIC	T3cN1M0	BSO	peritoneal metastasis, para aortic lymph node	-
51	71	IIIC	T3cN1M0	ATH + BSO + OM + rectal resection	peritoneal metastasis, para aortic lymph node	-
61	57	IIIC	T3cN0M0	ATH + BSO + OM + rectal resection	peritoneal metastasis	-

ATH: abodominal total hysterectomy, BSO: bilateral saipngo oophorectomy, OM: omentectomy, NAC: neoadjuvant chemotherapy

Supplementary Table S2: Sequence of siRNA used in the study.

Target gene	siRNA	Target sequence (5' to 3')
non-targeted	control	TTCTCCGAACGTGTCACGT
MITF	MITF-1817	TTCCTTTAATATGAAATTTTTTT
	MITF-2688	TTGTATTAATTTGTAAGAATATG
	MITF-2825	TTGCGAATATTTTGTAAATTACA
	MITF-3700	CAGTTTTATAGCTTTATTTCTTA

Supplementary Table S3: Sequence of primers used in the study.

target	primer	primer sequence (5' to 3')
GAPDH	GAPDH-Fw	CATGTTCGTCATGGGTGTGAACCA
	GAPDH-Rev	AGTGATGGCATGGACTGTGGTCAT
MITF	MITF-Fw	GGGCTTGATGGATCCTGCTTTGC
	MITF-Rev	GGTGGGGGCAGACCTTGGTT
MCAM	MCAM-Fw	GGCCGGCCTCTGAAGGAGGAGAAGA
	MCAM-Rev	CTGTGCCTTCAGAATACTCTGCAAGG
